# *I felt so much conflict instead of joy:* an analysis of open-ended comments from people in British Columbia who declined care recommendations during pregnancy and childbirth

**DOI:** 10.1186/s12978-021-01134-7

**Published:** 2021-04-15

**Authors:** Kathrin Stoll, Jessie J. Wang, Paulomi Niles, Lindsay Wells, Saraswathi Vedam

**Affiliations:** 1 Birth Place Lab, Department of Family Practice, University of British Columbia, 304-5950 University Blvd, Vancouver, BC V6T 1Z3 Canada; 2 Faculty of Medicine, University of British Columbia, 317-2194 Health Sciences Mall, Vancouver, BC V6T 1Z3 Canada; 3 Midwifery Education Program, Department of Family Practice, University of British Columbia, 304-5950 University Blvd, Vancouver, BC V6T 1Z3 Canada; 4 New York University Rory Meyers College of Nursing, 433 1st Avenue, New York, NY 10010 USA

**Keywords:** Respectful maternity care, Declining care, Refusal of care, Shared decision-making, Lived experiences, Care narratives, Informed consent, Childbirth, Person-centered care

## Abstract

**Background:**

No Canadian studies to date have examined the experiences of people who decline aspects of care during pregnancy and birth. The current analysis bridges this gap by describing comments from 1123 people in British Columbia (BC) who declined a test or procedure that their care provider recommended.

**Methods:**

In the *Changing Childbirth in BC* study, childbearing people designed a mixed-methods study, including a cross-sectional survey on experiences of provider-patient interactions over the course of maternity care. We conducted a descriptive quantitative content analysis of 1540 open ended comments about declining care recommendations.

**Results:**

More than half of all study participants (n = 2100) declined care at some point during pregnancy, birth, or the postpartum period (53.5%), making this a common phenomenon. Participants most commonly declined genetic or gestational diabetes testing, ultrasounds, induction of labour, pharmaceutical pain management during labour, and eye prophylaxis for the newborn. Some people reported that care providers accepted or supported their decision, and others described pressure and coercion from providers. These negative interactions resulted in childbearing people feeling invisible, disempowered and in some cases traumatized. Loss of trust in healthcare providers were also described by childbearing people whose preferences were not respected whereas those who felt informed about their options and supported to make decisions about their care reported positive birth experiences.

**Conclusions:**

Declining care is common during pregnancy and birth and care provider reactions and behaviours greatly influence how childbearing people experience these events. Our findings confirm that clinicians need further training in person-centred decision-making, including respectful communication even when choices fall outside of standard care.

## Plain english summary

Conflict between pregnant people and providers can ensue when there is a difference in opinion about the right care for the mother or newborn. In these situations, pregnant people retain the right to decline care they do not want. While much of the literature focuses on the experiences of healthcare providers when care is declined, very little is known about how childbearing people experience these interactions. The current paper addresses this gap by presenting findings from 1123 childbearing people in Canada who provided comments about their experiences of declining a test or procedure that their maternity care provider recommended. Results showed that declining care is common. The most common procedures declined were gestational diabetes testing, ultrasounds, induction of labour, pharmaceutical pain management during labour, and eye prophylaxis for the newborn. Respondents gave many reasons for declining care, with the most common being their belief that the test, medication, or procedure was not necessary, or did not align with their values. Childbearing people described three types of interactions with health providers when they declined care: being informed, feeling coerced to accept care, and losing trust. Feeling respected and heard and having all of the information to make decisions enhanced their comfort, but pressure to comply led to feelings of disempowerment and distrust. To avoid conflict and differences in opinion, care providers can ask about and understand the expectations, needs, fears, and preferences of pregnant people early in care and provide enough time for discussions about care options.

## Background

The current study took place in British Columbia (BC), the most Western province of Canada. Approximately 40,000 people give birth each year in BC. About half of childbearing people give birth under the care of obstetricians, 30% with family physicians and 15% with midwives [1]. Labour and birth nurses also provide intrapartum care at hospitals, together with the primary care provider. Home birth is an option for midwifery clients who prefer this setting and meet criteria for home birth. Midwives and family physicians in BC offer mostly community-based, caseload care and provide continuity of care.

The published literature about shared decision making (SDM) and informed choice is vast [2].Yuill et al. (2020) performed a meta-synthesis of 37 qualitative studies of women’s experiences of decision making and informed choice during pregnancy and birth [3]. The included studies described SDM and informed choice as complex and dynamic processes that are characterized by three themes (uncertainty, bodily autonomy & integrity and performing good motherhood) and three interlinking actions (information gathering, balancing choices and aligning with a birth philosophy). The results from the synthesis also highlighted the importance of family history and experiences during past pregnancies when making decisions.

Yuill et al. (2020) describe the disconnect between health care providers who believe they are offering women choices and childbearing women who feel that they have limited control and decision-making power. This has led some authors to describe SDM and informed choice as illusory, idealized or abstract concepts that do not translate well into practice [3]. For example, providers ought to respect the right of pregnant people to make informed choices and facilitate this process by providing complete, relevant, and objective information in a non-authoritarian, supportive manner. Pregnant people and their caregivers can then work together to make decisions [4]. In reality, numerous obstacles to implementing SDM in health care have been described [2] and several authors have identified the need for SDM to be more person-centered, through collaboration with patients, non-interference, getting to know the person and providing a context of care that supports autonomy. [5] When a pregnant person declines recommended treatment, or requests treatment that a care provider believes is unsafe, they retain the right to respectful care. However, these situations can cause conflict between a childbearing person and their caregiver and contribute to negative experiences and poor outcomes [4].

How pregnant people experience care when they disagree with their provider’s recommendations is not well understood. By analyzing open-ended responses from an online survey from the *Changing Childbirth in BC* Study [6, 7], we describe experiences of people who declined their care provider’s recommendations during pregnancy, birth, and/or the postpartum period. Increased knowledge in this area may assist care providers in supporting autonomous decision-making and providing respectful care to all pregnant people, regardless of their choices.

### Existing literature

The published literature on declining maternity care mostly focuses on care providers’ experiences [8–11]. Frameworks for practitioners to guide informed decision-making have been developed out of this research.

Cahill identified recurring issues of paternalistic and defensive practice with regard to providing patients with informed choices [12]. Clinical decision-making was based on physiological indicators, rigid adherence to protocols, poor communication and documentation, and failure to acknowledge people’s views and feelings. Lyerly et al. stated that the perception and communication of risk is an ongoing challenge in maternity care and can lead to care that is neither evidence-based nor patient-centered [13]. Ultimately, the goal of informed choice is that pregnant persons understand all of their options and are able to decide what is in their or their newborns’ best interest. Not only do paternalism and perceptions of risk influence many informed choice discussions, but care providers’ opinions and experiences have been prioritized over patient preferences and experiences within the literature. Two papers described midwives’ experiences when their clients declined standard care with no reference to the impact that this had on their clients [8, 11].

Very few studies identify reasons why pregnant people decline care recommended by their midwife, obstetrician, family physician, or nurse, or the impact that this has on them. Jenkinson and colleagues explored the experiences of people, midwives, and obstetricians when people declined recommended care [9, 14]. Specifically, researchers interviewed healthcare providers (n = 12) and childbearing people (n = 9) who received care at a tertiary hospital in Australia that instituted a process to document refusal of care. While care providers felt “protected and reassured by the structured documentation and communication process”, childbearing people and some midwives felt that risk discourse and pressure to accept unwanted care were still prevalent [14]. In another study, Chigbu and Iloabachie interviewed 62 Nigerian childbearing people postpartum to explore reasons for declining cesarean Sect. [15]. Reasons included fear of death, economic factors, desire to experience vaginal delivery, and inadequate counselling. These studies provide important but limited information about the phenomenon. They do not provide insight into the response of the healthcare system, how that response was interpreted by the service user, nor the ultimate impact on their  well-being.

Patient-provider interactions, access, respect, and self-determination are relevant in all health care arenas. Health systems have increasingly turned their attention to expanding access to person-centred care, and British Columbia has articulated a health quality framework that centers the importance of honouring people’s choices and optimizing maternal health and wellness [16] and a recent independent provincial investigation calls for the application of indicators that hold systems accountable by assessing human rights in health care. [17] Before the current study was conducted very little was known about the priorities of maternity care service users in British Columbia, and their experiences when declining care.

The current paper addresses these gaps by providing the most comprehensive analysis to date, based on a large dataset, on the types of tests and procedures that childbearing people decline, why they decline care, perceptions of their care providers’ reactions, and how they describe the impact on their quality of care, safety, and mental health.

## Methods

A Steering Council of 18 people with previous childbirth experiences and a few planning pregnancy collaborated with researchers to design *Changing Childbirth in BC*, a provincial community-led mixed methods study of people’s experiences while accessing pregnancy and birth care. Steering Council members represented various groups, including immigrants and refugees and those with a history of incarceration and housing instability. Prior to convening the Steering Council, our community partner (Midwives Association of BC) surveyed 1333 people across the province, who provided feedback about key areas for study. Two topics prioritized by those surveyed were: (1) *My birth experience* and (2) *My experience around how decisions were made during my pregnancy*.

Following a broad literature review to collect relevant validated items for a cross-sectional survey, the Steering Council members participated in a content validation process to assess relevance, importance, and clarity of items, adapting or creating new items when necessary. The final instrument collected information on demographics, preferences for and access to model of care, maternal and newborn outcomes, and 31 items describing preferences for and experiences of decision-making over the childbearing cycle, including experiences of declining care.

### Recruitment

Following approval by the University of British Columbia Behavioural Research Ethics Board (# H12-02418), the survey was made available through a public website, and all non-governmental organization (NGO) and community partners disseminated the link and information to people of childbearing age across BC between January and June 2014. The survey was advertised through posters and social media outlets, and reminder notices were sent by email, postcards, community listservs, and NGO websites. All institutional partner organizations, including a large provincial referral hospital and maternity clinics, recruited study participants. Clinician team members encouraged their colleagues to disseminate to their patients/clients. In the current paper, we report on survey responses from people who declined tests, treatments, or procedures at some point during their pregnancy, birth, or the postpartum period.

### Analysis

We conducted content analysis of open-ended responses to the question: *At any time did you refuse to accept any care that a nurse, doctor or midwife offered to you or your baby? "Care" includes anything that might be done or given to either of you or that you were asked to do (take a test, treatment, medicine, *etc*.).* People who answered “yes” were prompted to answer an open-ended follow-up question: *Please tell us what you refused, why you refused it, how the staff reacted, and how you felt about it. We would appreciate as much detail as you would care to provide.* People could answer this question for up to three pregnancies/births.

The analysis team included two midwife clinician researchers (SV, PN), medical (JW) and midwifery (LW) trainees, and a reproductive health researcher with a background in psychology and sociology (KS). We used quantitative content analysis [18, 19], to categorize and describe the qualitative data, and to identify a sample of comments for a more nuanced qualitative content analysis.

Given that comments were made in response to an open-ended survey question, and very little is known about the phenomenon, we chose to use conventional content analysis, an approach where categories emerge from the data rather than being guided by existing theory or literature. When using this approach text data is coded into categories that can then be described using statistics. [20] Specifically, one author (JW) performed line-by-line counting of tests and procedures that people declined, and coded reasons for declining care, care provider reactions, and childbearing people’s feelings about the situation into main and subcategories. Prior to analysis, three team members (SV, KS, JW) reviewed the first 200 comments and agreed on a preliminary list of data categories for each of the four elements of the question. This process was meant to enhance coder reliability. JW used this list to code the data, and added new categories as needed. For the last component of the question (childbearing people’s feelings about the situation), midwifery trainee LW reviewed the categories and subcategories and how they might be connected, to gain a deeper understanding of the experiences of childbearing people  who decline care. A qualitative researcher with clinical expertise (PN) provided oversight and review of this process to assist with developing meaningful interpretation of the findings.

Braun et al. (2020) [21] describe the benefits of collecting qualitative data via online surveys and different analysis approaches. Online surveys are a good way to collect data about sensitive topics and a convenient way for participants to provide data as they have more control over the process (e.g. when to participate). Analysis of qualitative survey data can range from descriptive to interpretative and discursive. Our team used a mix of description and interpretation in the current analysis.

SV and PN contributed insight on clinical relevance of the findings and data was analyzed using NVivo software. Three of the analysis team members are also parents with lived experience of declining aspects of maternity care or opting for care that was outside the norm (e.g. home birth).

## Results

### Sample characteristics

The *Changing Childbirth in BC* dataset included 2100 childbearing people who reported on 3586 pregnancy/birth experiences. People who answered the question about whether they refused any care were encouraged to describe their experiences using free text: 1123 people provided a total of 1540 responses, meaning that some people responded to the question for each of 2 or more pregnancy/birth experiences. Some responded with a few words, and others wrote one or more paragraphs. The unit of analysis is the pregnancy or birth experience associated with declining care.

The majority of people who reported declining care (89.3%) experienced their last birth between 2010 and 2014 (i.e. within 5 years of data collection), and all people reported on pregnancy/birth experiences in BC. Characteristics of people who provided comments about declining care and those who did not are reported in Table 1. The two groups are comparable on most characteristics with the exception of prenatal care provider and mode of birth. People who provided comments about declining care were more likely to receive care from midwives and less likely to give birth by Caesarean.

**Table 1:** Characteristics of participants (n = 2100)

	Provided comments about declining care (n = 1123)	Did not provide comments about declining care (n = 977)
Number of pregnancies (mean)	2.2	2.1
Number of children (mean)	1.7	1.6
Age at time of data collection (mean)	32.7	33.0
Identified with a vulnerable group*	90 (8.0)	70 (7.2)
Ethnicity Asian White Other Missing	26 (2.7) 875 (92.1) 49 (5.2) 173	32 (4.5) 655 (92.1) 24 (3.4) 266
Highest level of education is Highschool	97 (10.1)	67 (9.4)
Family income $ 30,000 or less	75 (6.7)	47 (4.8)
Care provider during pregnancy Family Physician Midwife Obstetrician Other Multiple responses possible	225 (20.0) 883 (78.6) 208 (18.5) 82	313 (32.0) 539 (55.2) 259 (26.5) 92
Gave birth by Cesarean section	184 (17.2)	212 (24.5)

*Identified as one or more of the following: family income < 30 k, immigrant or refugee, history of incarceration or housing instability

### What types of tests and procedures did people decline?

**Fig. 1:**
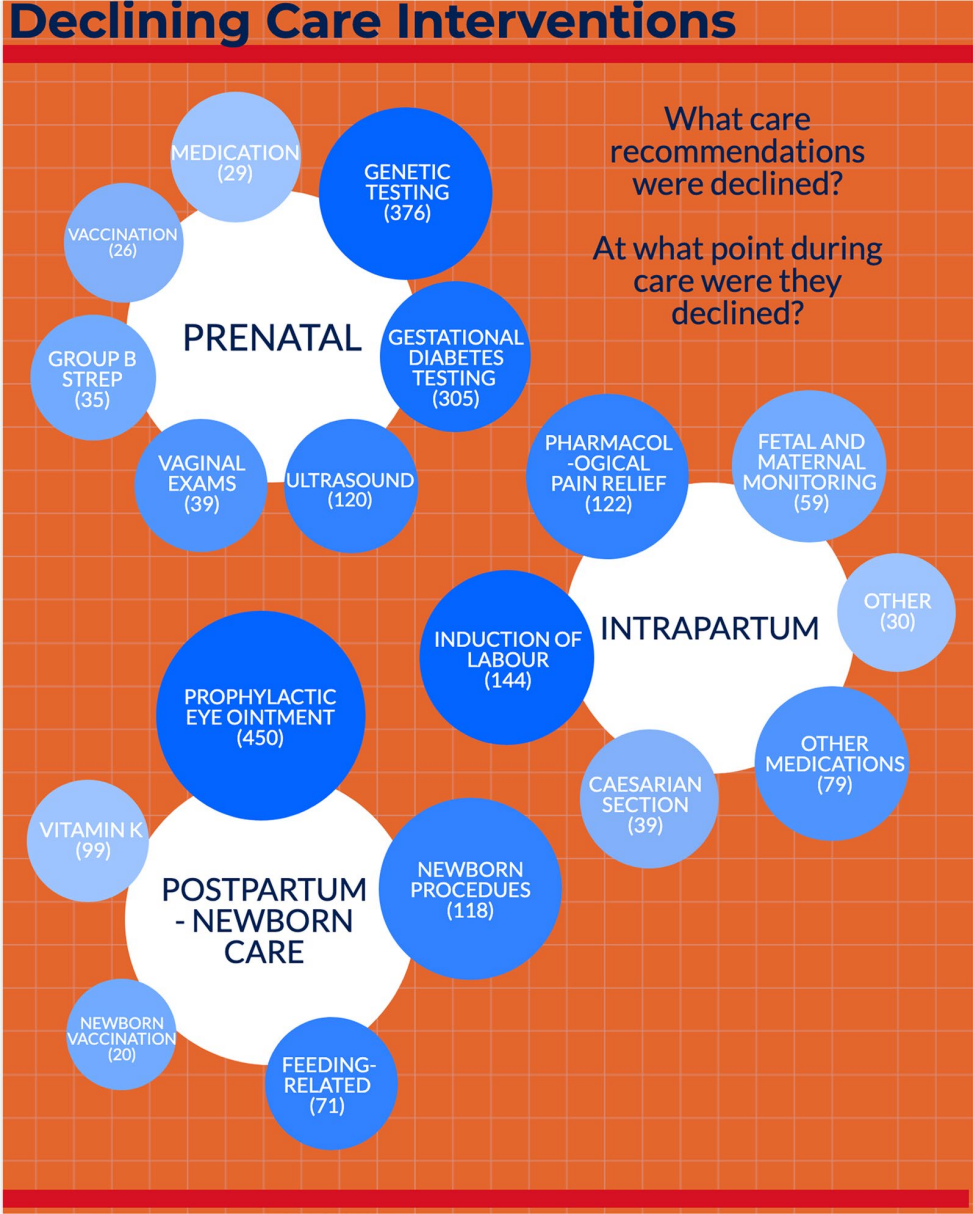
Types, timing, and frequencies of tests and procedures that were declined

A total of 2478 comments referred to tests, medications, and procedures that people declined (see Fig. 1). During pregnancy, the most commonly declined assessments were genetic testing (e.g. amniocentesis, chorionic villi sampling), gestational diabetes testing, and ultrasound(s). During labour and birth, participants most often declined induction of labour (e.g. membrane sweeping, oxytocin, etc.), pain relief (e.g. nitrous oxide, epidural etc.), fetal or maternal monitoring, and medications (e.g. antibiotics, rhogam, magnesium sulfate, anticoagulation, castor oil, etc.). The pain management method most often refused was epidural (40 comments), and the medication most often declined was antibiotics (48 comments). In the postpartum period, the most commonly declined procedure was application of eye ointment to the newborn (erythromycin antibiotic prophylaxis), followed by vitamin K for the newborn, and selected procedures, such as cord clamping (immediate or delayed), suctioning, in-hospital bathing, baby foot printing, newborn metabolic screening via heel blood draw, circumcision, mother-baby separation, hearing screen, etc.

### Why did people decline tests and procedures?

There were 1366 comments that described why tests or procedures were declined. Responses could be categorized into 9 categories, the most common of which were that the participant felt that the test, procedure, or medication was **unnecessary** (572 comments) or did not align with the **person’s values** (303 comments); they **preferred an alternative** (135 comments) or considered the test/procedure **bad for baby or unsafe** (104 comments); or they had **access to information** or had reviewed research that did not support use of the test, medication, or intervention (86 comments). Less commonly reported were refusals because they **felt uncomfortable**, because of **health reasons**, because **healthcare providers were being rude or incompetent,** or because they felt that it was too **inconvenient.** Some comments fell into the **Other, Unspecified** category. Categories and illustrative quotes are listed in Table 2.

### How did care providers react?

Care provider reactions were described in 1414 comments. The majority (603 comments) were about care providers **accepting the decision,** and 373 comments were about healthcare professionals **supporting the decision.** However, 180 comments referred to care providers **reacting with disrespect** in response to the refusal*,* or providers **trying to convince them** to accept care they did not want (158 comments). Some comments (72) were about care providers not accepting or honouring the decision and **proceeding without consent.** Finally, 28 comments could not be categorized and fell into the **Other category.** Categories and illustrative quotes are listed in Table 2.

**Table 2:** Reasons people declined tests or procedures, with illustrative quotes

Why did people refuse tests and procedures?	Illustrative quotes
Unnecessary (n = 572)	“I did not see the need to” “I did not have risk factors” “I later accepted the intervention when it became necessary”
Did not align with the patient’s values (n = 303)	“I did not want one” “I would not terminate pregnancy anyways” “I wanted to labour naturally”
Preference for an alternative (n = 135)	“We opted for oral vitamin K” “I decided on forceps instead” “I kept a healthy diet instead of taking insulin”
Considered bad for baby (n = 104)	“I did not want goop in my baby’s eyes” “The risks of amnio are too high”
They had access to information or had reviewed research that did not support the medication/intervention (n = 86)	“The research says the intervention does not improve outcomes” “I heard that the test is inaccurate” “The false positive rate is too high”
They felt uncomfortable (n = 38)	“I have a strong fear of needles” “Putting in an epidural is too painful”
Health reasons (n = 31)	“I have a suspected allergy to analgesics” “Laughing gas made me feel sick”
They felt that healthcare providers were being rude or incompetent (n = 28)	“The doctor was very pushy” “The nurse was incompetent” “I refused to see that OB again”
They felt it was too inconvenient (n = 14)	“I have a toddler at home” “I was busy with work”
Other, Unspecified (n = 55)	“I didn’t like it the last time” “I was abused in the past”

### How did childbearing people feel about the situation?

There were 341 comments in this section that could be grouped into three categories: (1) being informed, (2) losing trust and (3) feeling pressured (Table 3).

**Table 3:** Care provider reactions, with illustrative quotes

How did care providers react?	Illustrative quotes
Provider accepted the decision (n = 603)	“The nurse respected my decision” “They were fine with it”
Provider supported the decision (n = 373)	“I felt supported” “My midwife was very supportive” “The doctor presented the pros and cons and left the decision up to us”
Provider did not react respectfully (n = 180)	“The nurses seemed offended” “They clearly thought I was making the wrong decision” “He belittled me”
Provider tried to convince them to accept care (n = 158)	“I felt very pressured” “He tried to scare me into taking insulin” “She pulled up all these scary stats”
Provider did not honour decision and proceeded anyways (n = 72)	“She did it without my consent” “I asked for delayed cord clamping but they cut it right away” “I screamed for her to stop and she just kept going” “She fed the baby behind my back”
Other (n = 28)	“I don’t recall their reaction”

#### Being informed

Participants reported that informed choice discussions and feeling respected enhanced their care experience. In situations where care was declined, participants stated that feeling validated and respected led to the experience of *“feeling informed”*. Participants also appreciated having both knowledge and choice in deciding what was right for them and their family. One participant wrote: *“I didn't really feel like I was refusing treatment so much as making a choice.”* The link between information and choice was vital, and it framed choices around care as an *“offer”* rather than being prescribed or ordered. This approach led to clients feeling in control of their care. One participant wrote:

“[…] We discussed these topics, I asked questions and expressed my concerns, she addressed my concerns directly and then left the decision up to me. I felt she provided me with enough information to make my own decisions and that she trusted me to do so. I really appreciated that my midwife never seemed nervous or afraid of my decisions, and never tried to intimidate or influence me by emphasizing or exaggerating risk factors […]”

When care providers supported person-led decision-making, by offering information and options, “refusing” care was viewed as a choice rather than a refusal. One person observed:

“[…] since everything was presented as options to be considered and decided upon, it didn't feel like I was 'refusing to accept care' when I said no to things. I did say no to a number of things...like prenatal testing, eye gel for the newborn, some of the gestational diabetes guidelines I was given.... but none of that felt like refusing care, it was just part of the care I received while pregnant [...]”

#### Being pressured

While the above participants described situations of being in control of their care decision, others felt dissatisfied and disappointed in their care experiences. Pressure to conform, combined with a state of vulnerability, led to these feelings of disempowerment. A predominant experience of *“feeling pressured to give in”* arose. Participants used language such as “*persuaded”, “ganged up on”, “coerced”, “badgered”, “forced”, “pushy”, “convinced”, “submitted”,* and *“insisting”* to convey how their decisions were received by providers. Participants described that they *“felt disappointed about feeling that pressure”*.

Childbearing people recounted these as forms of coercion or pressure that were imposed upon them by healthcare providers. Some participants found themselves in a vulnerable position and ultimately relented, giving in to doing things they did not want. When reflecting on this vulnerable state and the situations that occurred, some described being disappointed in themselves:

“[I] refused formula in the hospital when having challenges learning how to breast feed. I didn't want my baby to receive formula. Staff respected [me] in that moment but came back multiple times with pressure to accept formula every time after we would try breast feeding. Eventually my husband agreed to give formula in a moment where I was feeling too emotionally drained to keep refusing. I felt like a failure. Like I couldn't give birth properly and couldn't feed my baby properly either.”

Some participants described treatment by healthcare providers that made them feel humiliated and powerless, akin to torture:

“I did not want to lie on my back or go in an ambulance but was forced to do both via physical force. I was also tied down in the ambulance which I did not want and which felt like torture. Also my midwife kept exposing my buttocks and privates in the ambulance and I felt humiliated as there was a male ambulance attendant right there. I do not understand why this was done and it made me feel completely powerless and humiliated.”

A small number of participants described the trauma caused by feeling coerced:

“I had been told about episiotomies prior to labour, and was clear that I didn't want one. I thought that my doctor understood, as she didn't pursue it. However, during the delivery she said she was going to give me the episiotomy. I refused. She said it was a routine procedure to prevent tearing. She did it without my consent. During the episiotomy I screamed out for her to stop, that I could feel her cutting. She told me that was impossible and kept going. The pain was extreme…I felt traumatized by the whole birth. I subsequently avoided gynaecological exams for years…”

In an extreme case, another participant described how the experience of coercion in a prior birth led to their decision to give birth unassisted:

“[…] The midwife and the nurse kept applying fetal monitors even though I was throwing them off and yelling "NO, NO, NO". By this time I had been on pitocin for 10+ hours, it had been 2 days since I last ate, and all the pain meds had worn off. They kept trying to get me to agree to a c-section and told me I would be allowed pain meds if I agreed, and that I could eat afterwards too. It was a horribly disgusting abuse of power. It is totally unacceptable that people are treated this way. I am currently pregnant and am planning an unassisted birth because I refuse to go back to the hospital and be battered and abused again - with NO recourse available and no accountability whatsoever. I will not pretend that it's okay to treat people like that. Baby and I will be safer at home […]”

#### Losing trust

Interactions of a paternalistic nature often lacked information and consent, which contributed to an overall mistrust of care providers. Some chose to decline recommendations if they felt under-informed about the decisions they were making, leading to a perception of negative care experiences. One participant wrote: *“…[the doctor] came in telling me WHAT was going to go on with my labour, rather than asking what my partner and I wanted to do, and explaining the options.”* Participants did not appreciate feeling like they lacked information and decision-making power during their care. This is evident in another participant’s story:

“I refused to be induced at 38 weeks because they never had any solid reasons for wanting to induce me, and kept talking about this very early on (prior to 30 weeks). First they said baby would be too big, and so they wanted to induce. Then they said baby would be too small, and so they wanted to induce. They never stated what made them think this, or why, and I refused to consent to that. I said that we would wait until 40 weeks and THEN we could start discussing it, if it was needed.”

Disrespect or disregard for the wishes of people also generated mistrust. When they felt that their decisions were not respected and supported by their care provider(s), one participant recounted making the decision to discontinue care:

“I was diagnosed with gestational diabetes at about 34 weeks gestation with my twins. I tried using insulin to control my blood sugars but felt terrible when on the insulin and chose to stop treatment. The endocrinologists and diabetes clinic nurses at [name of hospital] were very aggressive in pushing for treatment with insulin and actually played down some of the risks/dangers associated with insulin usage. They could not provide me with any other options other than I HAD TO USE THE INSULIN, to the point where I was told my twins would be premature, have immature lung development, and low blood sugars at birth. I stopped going to the clinic and using the insulin treatment and tried controlling my dietary sugar intake. My twins were delivered on their scheduled date (at term) and had no health problems at birth and currently.”

In another account, the participant reported that their needs and wishes were disregarded, which led to loss of trust in the midwife and stress for the client:

“[…] Although my second pregnancy was over a decade later, emotionally I needed to have as few people as possible involved. The midwife tried to persuade me to allow the practicum student. I remained clear that this was my pregnancy and that I had to have my need for privacy respected. When I next showed up for an appointment, there was a practicum student waiting in the room. I respectfully reminded the midwife that I had chosen not to have a practicum student involved. The midwife said that she was here now, so couldn't she just stay. I said sorry, but no. She said that the practicum student came all this way for nothing. Again, I said sorry, but no. The practicum student left, but the midwife was visibly displeased. I felt stressed by this incident, and it negatively impacted my trust and respect for the midwife […].”

Several participants described experiences of losing trust in care providers when they were asked to do things that contradicted what they had learned about labour and birth. Often participants described situations in which medical interventions were pushed on them that they thought to be unnecessary. The person below describes how questioning a health care provider can result in being labelled a difficult patient.

[…] [I] Refused to labour on my back, as the assessment room nurse requested. She wanted me to do this because I had continuous EFM and the leads kept losing the heartbeat due to my moving. I was having an unmedicated labour, and wanted to move around, but couldn't get out of bed because I was in the [name of hospital] assessment room […]. I think I said, "are you joking?" and continued to roll onto my side to labour. I felt confident in my decision, but lost a bit of faith in her skills as a nurse that she would ask me to labour on my back. The continuous EFM was just a precaution due to meconium, and the baby's heartbeat had been normal the entire time. She seemed to accept this, although I did feel that she treated me a bit like a difficult patient after that. […]

## Discussion

This is the first study in Canada to explore the experiences of childbearing people declining care offered or recommended by midwives, physicians or nurses. In our sample, more than half of participants declined care at some point during pregnancy, birth, or the postpartum period (53.5%), making this a common phenomenon. The most commonly declined test or procedure was prenatal testing, such as genetic or gestational diabetes testing, and newborn treatments (eye ointment for the newborn, vitamin K). Declining tests or procedures during labour or birth was less common. Participants described many reasons for declining care, but the most commonly cited were beliefs that the test, procedure or medication was unnecessary or did not align with their values.

A study with maternity care providers in the Netherlands, about maternal requests that go against medical advice, revealed that women most frequently declined gestational diabetes screening (66.3%), hospital birth (65.3%), and fetal monitoring (both continuous and intermittent) during labour (39.6%) [22]. These results align with the findings of the current study and warrant further exploration of how care providers can best communicate the rationale for these tests and procedures to clients and remain respectful when clients decline care.

Our finding that childbearing people most often decline care during pregnancy is supported by research with more than 2000 pregnant and postpartum people in the United Kingdom, who were surveyed about their ability to exercise informed consent. Perceptions of informed choice were very different for tests/procedures in the prenatal period compared to birth. For instance, 73% reported making an informed choice about genetic screening during pregnancy, but only 31% felt they made an informed choice about electronic fetal monitoring (EFM) during labour [23]. The best available evidence shows that continuous EFM versus intermittent monitoring is associated with an increase in Cesarean sections and instrumental vaginal deliveries but no decrease in neonatal mortality [24]. Hersh et al. have published a case report that describes how care providers can support decision-making around intermittent auscultation by using a woman-centered decision- making pathway for fetal monitoring [25].

These results, together with findings from the current study, emphasize the need for care providers to begin discussions during pregnancy about the pros and cons of common labour and birth interventions and procedures, including the evidence basis for recommendations, so that people have time to understand different procedures, and an opportunity to consider their options.

When participants recounted care provider reactions to their decision to decline care the majority said that their decision was accepted or supported; however, a sizable number also described care providers who were disrespectful or pressured them to accept care they did not want. At times, participants questioned if they were doing the right thing leading to perceptions of conflict rather than joy as described in the title of this paper.

In the current study, feeling pressure from maternity care providers led to feeling vulnerable and invisible and resulted in loss of autonomy. These findings suggest that both what is offered and the way it is being communicated are equally valuable to childbearing people. Being pressured into complying with unwanted care can have long-lasting psychological consequences and can lead to termination of care, as our results indicate. In one study with more than 1500 people in the United States (US) who recently gave birth at a hospital, predictors of birth-related post-traumatic stress disorder (PTSD) were assessed. Pressure from care providers to have an induction or cesarean section was one of the factors significantly linked to PTSD symptoms [26]. In an analysis of the full *Changing Childbirth in BC* dataset Vedam et al. [27] identified that pressure to have an induction or cesarean section, along with perceived differences in opinion between childbearing people and their providers (about care options), were significantly linked to loss of autonomy among childbearing people. These findings, together with results from the current analysis, establish a firm connection between pressure and coercion from care providers, loss of autonomy, and post-traumatic stress [22].

Our findings show that failure to gain clear, unambiguous consent for procedures or treatments contributes to mistrust of care providers among people who decline care. Feeling betrayed and powerless and losing trust in care providers were also themes that emerged in the stories of 40 women who described their births as traumatic [28]. In another study with 2192 women from the Netherlands, loss of control and fear for the baby’s life were the two most commonly reported reasons why people felt traumatized. When asked what care providers could have done to prevent the traumatic experience, the most common answers were: 1) communicating more and explaining things better, 2) listening more, and 3) providing emotional support [29]. Our findings support the results from the Netherlands, as many participants noted that care providers did not listen and/or did not take the time to explain if and why a test or procedure was necessary.

Reed et al. surveyed 748 women in Australia who described a traumatic birth experience [30]. Common themes included “prioritising the care provider’s agenda”, “disregarding embodied knowledge”, “lies and threats”, and “violation”. These themes resonate through our data as well, with many accounts of care providers ignoring people’s knowledge of their own bodies and/or rigidly promoting care plans that people did not agree with.

Negative birth experiences and birth trauma impact the transition to parenthood negatively [31] and are linked to decisions to avoid contact with the healthcare system in future pregnancies [32].

Midwives, family physicians, obstetricians, and nurses can provide respectful maternity care by ensuring client autonomy is supported through engaging in a person-centered decision-making process [33]. However, research with maternity care providers shows that they support people’s right to make decisions about their own care “within reason” and that the wishes of pregnant people can be overridden during emergencies [34]. The higher risk of mistreatment during emergency situations has been demonstrated from the perspectives of childbearing people. In the *Giving Voice to Mothers* study, 2700 childbearing people were surveyed about pregnancy experiences in the US between 2010 and 2016. People with emergency cesarean sections reported higher rates of mistreatment by care providers, including pressure to accept treatment they did not want, compared to those with a vaginal birth or planned cesarean Sect. [35].

In the current study, participants described such interactions as “abuses of power” and were distressed by these care provider behaviours. Morton et al. surveyed close to 300 maternity care providers in the US and Canada and found that they often witnessed verbal abuse, specifically threats to the baby’s health and well-being if the pregnant person did not comply with recommendation [36]. Our results support Morton’s findings, as several participants described being told that their newborn was in danger, with the implication that they were unfit mothers for not complying with recommendations. Bioethicist Raymond de Vries describes this phenomenon as the invisible mother, because “concerns and needs of women in labour fade in the face of hospital policies and the perceived needs of their soon-to-be-born babies” [37]. Other authors have grounded their analyses of situations where childbearing people decline care in critical feminist theory and highlighted how risk discourse and the dominance of medical knowledge are used to restrict or remove women’s bodily autonomy and right for self-determination [9].

The data also suggests that childbearing people in our sample declined tests or procedures because they found them to be unnecessary, preferred an alternative or had access to information that did not support use of the test, medication, or intervention. In some situations where people declined tests or procedures, care providers presented information with the sole purpose of gaining compliance, rather than discussing options. It is well accepted that knowledge is power and that there are inherent power imbalances in the provider-patient relationship [38]. In one study with 22 self-identified women of colour who were interviewed about their pregnancy and birth experiences, themes of power and control emerged. Specifically, respondents felt that care providers were controlling the information that women received, and how information was provided affected the level of autonomy and self-determination of childbearing women. Information provided by healthcare providers that was perceived as truthful, comprehensive and unbiased supported autonomous decision-making whereas information that was withheld, misleading, or biased reduced autonomy. Participants also noted that the way care providers communicated information depended on women’s ethnicity, educational level, insurance status and other factors and that a trusting relationship with care providers enhanced women’s experiences with care [38].

### Alignment of findings with meta-synthesis of SDM and informed choice

We consider our results in the context of themes and interlinking actions discovered during the meta-synthesis by Yuill et al. [3]. In the meta-synthesis childbearing women wanted to protect their bodily autonomy and integrity through making decisions about their care. When care providers denied this bodily autonomy childbearing people experienced loss of control. Our findings strongly resonate with this theme as many participants described the act of declining care as a way to control and protect their care experiences and their babies. Descriptions of denial of autonomy by care providers highlight the consequences of this denial, including loss of trust, and emotional reactions ranging from being disappointed to devastated and traumatized. Several participants recounted how previous negative or traumatic experiences during pregnancy and/or birth strengthened their resolve to reclaim control and autonomy by declining care they did not want in subsequent pregnancies, even planning to give birth unassisted.  The third theme ‘Performing good motherhood’ is also evident in our analysis because care providers at times tried to gain compliance by telling women that they were putting their babies at risk. The current analysis expands on the description of this theme by showing what happens when women are coerced to comply with care recommendations. For example, the midwifery client with breastfeeding challenges who repeatedly declined formula but gave in eventually because of exhaustion felt like a failure for not being able to breastfeed. In this situation, the woman’s internal perception of being a good mother was disturbed by giving formula to her baby. In other words, coercing women into accepting care or interventions they do not want can impact women’s internal perceptions of self-efficacy and motherhood.

The current analysis also shows strong support for the interlinking actions of information gathering, balancing choice and birth philosophy. Analysis of comments about why people declined tests or procedures clearly indicated that this decision was based on information gathering and/or birth philosophy. For example, 600 comments referred to people declining care because they thought the test or procedure was unnecessary. This strongly implies they either had gathered information that supported that decision and/or were committed to a low-intervention approach to labour and birth. Many others declined care because they preferred an alternative or had information that was contrary to care provider recommendations, enabling them to feel confident in the decision to decline care. Comments indicated that the balancing of risks and benefits and the managing of uncertainty were things that women mostly accomplished prior to declining care.

### Implications for practice

#### Informed choice

Childbearing people who trust their care providers are more likely to accept tests and procedures and feel more comfortable discussing reasons why they prefer to decline care [39]. Michelle DeBaets recommends ongoing discussions before and during birth, to establish a birth partnership that is focused on trust, two-way communication, mutual education, and person-centred decision-making. Care providers are encouraged to share their own birth philosophies and practices with clients, and extending the length of prenatal visits to allow them to develop an understanding of the preferences and expectations of clients [39]. A central feature of the birth partnership is mutual education about choices and the “values that inform those choices” [39]. The author provides a helpful set of questions that can guide these discussions (e.g. What are the person's core values and goals of birth? What are their fears? Are there specific forms of treatment that the person does or does not want? Why? If labour does not go as expected, how will the person address their options for interventions?) Childbearing people retain the right to refuse care recommendations and care providers can avoid conflict by building trust through respectful interactions, providing high-quality evidence and discussing options ahead of time. The author also offers important guidance for teams with differences in birth philosophy and practices, so that they can provide consistent and respectful care to clients [39]. 

Childbearing people from minority communities often experience systemic racism and/or discrimination when accessing health care, and as a result might be hesitant to trust providers. [17, 38]. Racial congruence between pregnant people and providers creates a shared understanding that helps to build trust. [38].

#### Health professional education

**Fig. 2:**
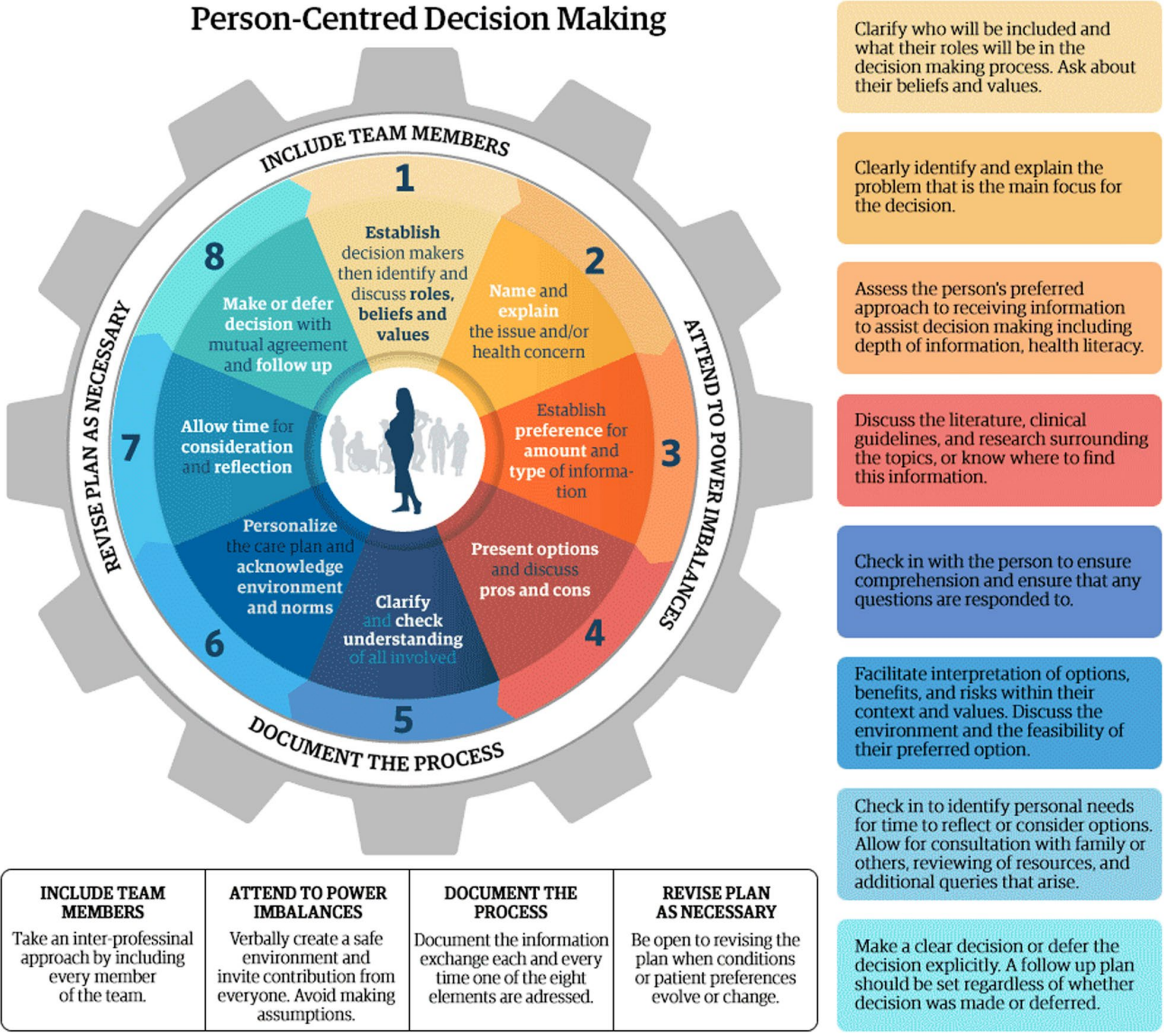
Graphic summary of *Dialogue and Decisions,* an online interprofessional course that teaches health professional learners a systematic approach to person-centred care

Efforts to identify and reach consensus about quality criteria and professional competencies for applying person-centered decision-making in maternity care have been published [40]. Several training programs in shared decision-making and person-centered decision-making for healthcare providers exist. For example, *Dialogue and Decisions* [41] is an online interprofessional course that explores the value and complexity of human interactions around healthcare decisions and teaches health professional learners a systematic approach to person-centred care (see Fig. 2). Case-based activities, exploring patient preferences and controversies around birth care, develop professional skills that enhance patient experiences of care. Participants learn best practices and evidence-based strategies to promote respectful communication, tranform conflict, and collaborate to provide person-centred care. Legare et al. identified 54 similar programs, including case-based discussion, small group educational sessions, roleplay, printed educational material, and audit and feedback [42].

### Health human rights

Findings from the current study also highlight the need for clear guidelines for providers around situations where pregnant people decline care. Some clinicians recommend having a second healthcare provider counsel the client, and documenting the informed refusal, while reassuring the client that they will continue to receive courteous, professional care regardless of their choice [4]. While these recommendations provide a good framework, it should be noted that our data suggest that involving a second care provider can be perceived by childbearing people as being “ganged up on” or being pressured to comply with recommendations, which might disrupt the care alliance between the childbearing person and their primary care provider. More importantly, Reed et al. note that women who felt bullied and coerced by care providers are more likely to report birth trauma. [30] These risks should be taken into consideration when care providers decide to involve other health professionals or family members in situations where care is declined.

Our findings confirm that clinicians need further training in supporting informed choice, and greater knowledge about health human rights [43] when clients make choices outside of standard care. Framing disrespect and abuse in response to people declining recommendations as human rights violations and gender-based violence [43] can raise awareness about the severity of these issues.

Finally, Jenkinson et al. propose a comprehensive, systems-level framework for documentation and communication with the goal of supporting people, clinicians, and health services in situations of maternal refusal [44]. Such frameworks centre the promotion of respectful maternity care, as described by Downe et al. [45]. While proactive strategies as described by DeBaets are best [39], opportunities for self-reflection and debriefing after negative encounters are important. This may provide an opportunity for people to process their labour and birth experiences, as well as for care providers to understand how disrespectful care affects childbearing people and families. Integrating respectful maternity care policies and practices into hospital settings takes time, and long-term success depends on both frequent engagement with key stakeholders and systems- and structural-level investments [45].

### Limitations

The findings of this study are based on a convenience sample of childbearing people who declined maternity care recommendations in British Columbia, Canada. The sample included few people of colour and a higher proportion of midwifery clients than utilization of care by childbearing people in BC. In a study with US women, equal proportions of people of colour versus white people declined care during pregnancy or birth but white people were more likely to report that their decision was respected. [46] A sample with a higher proportion of people of colour would likely show higher rates of disrespect from care providers in response to declining care recommendations. Our study also shows that midwifery clients are more likely to decline care and a sample with a higher proportion of people under the care of physicians would likely show lower rates of declining care.

In addition, the experiences of pregnant people may be different in other provinces or countries where maternity care systems and care options are different. Another limitation of this study is that we had a single coder for the qualitative content analysis of the 1540 responses. To mitigate bias, three team assessed coder reliability for the first 200 responses to seek consensus in data categories, the final coding was guided by an experienced qualitative researcher, and final coding and the results of the analysis were reviewed and confirmed by team members with lived experience of declining care recommendations.

Future research on this topic ought to include a more ethnically diverse sample of childbearing people and add questions about what tests and procedures people wanted to decline rather than did decline.

## Conclusions

Declining medications, procedures, and interventions is common during pregnancy, childbirth, and the period after birth, and care provider reactions and behaviours greatly influence how childbearing people experience these events. Those who report a positive birth experience felt supported and respected in their right to choose, whereas loss of autonomy, mistreatment, disrespect, pressure, and coercion from care providers were reported to have negative and long-lasting impacts on childbearing people. Translating these dimensions of quality into improvements at the point of care is challenging, but frameworks on how to respectfully support informed choice and refusal of standard care have been developed, [2, 10, 11] and health professional curricula are emerging.

## Data Availability

Study participants did not consent to have their data shared with others outside the study team. In addition, the comments include information that might potentially identify respondents or their care providers.

## Abbreviations

BC: British Columbia
NGO: Non-governmental organization
PTSD: Post-traumatic stress disorder
US: United States
